# A Phosphatidic Acid (PA) conveyor system of continuous intracellular transport from cell membrane to nucleus maintains EGF receptor homeostasis

**DOI:** 10.18632/oncotarget.9685

**Published:** 2016-05-31

**Authors:** Karen M. Henkels, Taylor E. Miller, Ramya Ganesan, Brandon A. Wilkins, Kristen Fite, Julian Gomez-Cambronero

**Affiliations:** ^1^ Center for Experimental Therapeutics and Reperfusion Injury, Brigham and Women Hospital and Harvard Medical School, Boston, MA, USA; ^2^ Department of Biochemistry and Molecular Biology, Wright State University Boonshoft School of Medicine, Dayton, Ohio, USA

**Keywords:** mammalian cells, breast cancer, gene expression, cell signaling, phospholipids

## Abstract

The intracellular concentration of the mitogen phosphatidic acid (PA) must be maintained at low levels until the need arises for cell proliferation. How temporal and spatial trafficking of PA affects its target proteins in the different cellular compartments is not fully understood. We report that in cancer cells, PA cycles back and forth from the cellular membrane to the nucleus, affecting the function of epidermal growth factor (EGF), in a process that involves PPARα/LXRα signaling. Upon binding to its ligand, EGF receptor (EGFR)-initiated activation of phospholipase D (PLD) causes a spike in intracellular PA production that forms vesicles transporting EGFR from early endosomes (EEA1 marker) and prolonged internalization in late endosomes and Golgi (RCAS marker). Cells incubated with fluorescent-labeled PA (NBD-PA) show PA in “diffuse” locations throughout the cytoplasm, punctae (small, <0.1 μm) vesicles) and large (>0.5 μm) vesicles that co-localize with EGFR. We also report that PPARα/LXRα form heterodimers that bind to new Responsive Elements (RE) in the EGFR promoter. Nuclear PA enhances EGFR expression, a role compatible with the mitogenic ability of the phospholipid. Newly made EGFR is packaged into PA recycling vesicles (Rab11 marker) and transported back to the cytoplasm and plasma membrane. However, a PLD+PA combination impedes binding of PPARα/LXRα to the EGFR promoter. Thus, if PA levels inside the nucleus reach a certain threshold (>100 nM) PA outcompetes the nuclear receptors and transcription is inhibited. This new signaling function of PLD-PA targeting EGFR trafficking and biphasically modulating its transcription, could explain cell proliferation initiation and its maintenance in cancer cells.

## INTRODUCTION

Epidermal growth factor (EGF) is a key contributor to cell growth, proliferation and differentiation by binding to its receptor, epidermal growth factor receptor (EGFR) (ErbB1), a transmembrane tyrosine kinase that initiates a signal transduction cascade and ultimately promotes cell proliferation, motility and invasiveness of cancer cells [1–4]. EGFR is localized to the plasma membrane and is then concentrated in vesicles following endocytosis [5]. Autophosphorylation of EGFR on specific tyrosine residues activates PI3K/AKT, Ras/MAPK, and JAK/STAT signaling pathways that lead to cancer cell survival [6]. The expression of EGFR is highly regulated in normal cells, whereas some cancer cells have high constitutive levels of EGFR. Understanding naturally occurring ways of downregulating EGFR in cancer cells is under intense investigation.

MTLn3 cells are a prime example of cancer cells with upregulated EGFR and currently uses as an excellent cell model for invasion [7]. Highly invasive metastatic breast cancer cells tend to possess larger calculated numbers of EGF receptors per cell than that of less invasive breast cancer cells or even normal cells [8–11]. EGF-stimulation in MTLn3 cells stimulates lamellipodia extension and increases actin nucleation, filament numbers and cell attachment to certain matrix proteins [12, 13]. Additionally, MTLn3 cells experience increased EGFR expression following agonist stimulation, which results in increased tumor cell migration with concomitant increases in intravasation and metastasis [4]. An EGF/CSF-1 paracrine loop has been documented that requires reciprocal signaling and chemotaxis between both cancer cells and macrophages for motility and invasion of MTLn3 breast cancer cells [14].

Phospholipase D (PLD) catalyzes the hydrolysis of phosphatidylcholine to generate choline and phosphatidic acid (PA). The physical properties of PA influence membrane curvature and allows for PA to act as a signaling lipid by recruiting cytosolic proteins to appropriate membranes [15]. Overexpression of PLD2 leads to an increase in *de novo* DNA synthesis and has been shown to regulate cell motility and proliferation [16–19]. PLD is a survival signal for serum-starved cells and induces the phenotypic change from ER+ to ER-, which corresponds with increased tumor invasiveness [20–23]. Inhibition of PLD-derived PA production retards cell migration of adenocarcinoma cells [20, 24]. PLD2 and EGFR form a heterodimeric complex that is mediated by the intracellular part of the receptor, which is independent of kinase activation [25].

Although PA targets a great variety of proteins in the cell, its concentration in mammalian cell membranes is maintained at low levels through the activity of several metabolic enzymes (such as phosphatases, phospholipases and kinases) [26, 27]. PA can be synthesized endogenously *in vivo* or added exogenously *in vitro* and is used as the backbone to generate other phospholipids [28]. Metabolic enzymes convert PA into diacylglycerol (DAG) very rapidly, and because DAG is the precursor for so many other lipids, it too is soon metabolized into other membrane lipids [29, 30]. This means that any upregulation in PA production can be matched over time with a corresponding upregulation in LPPs and in DAG-metabolizing enzymes. PA acts as a signaling lipid, recruiting cytosolic proteins to appropriate membranes, such that the physical properties of PA influence cell membrane curvature [31–33]. How temporal and spatial occurrences in the cell's compartments with the target protein are mediated is not fully understood given the transient nature of PA on both its intracellular levels and its physiological actions.

We found that intracellular PA governs the intracellular transport of target proteins. A “PA conveyor” exists that transfers PA from the cell membrane to the nucleus and vice versa and shuttles EGFR around the cell *via* PA vesicles. We also report that the positive and negative regulation is mediated by the nuclear receptors PPARα and LXRα.

## RESULTS

### EGFR and PLD2 are maintained in a feedback loop

**Figure 1:**
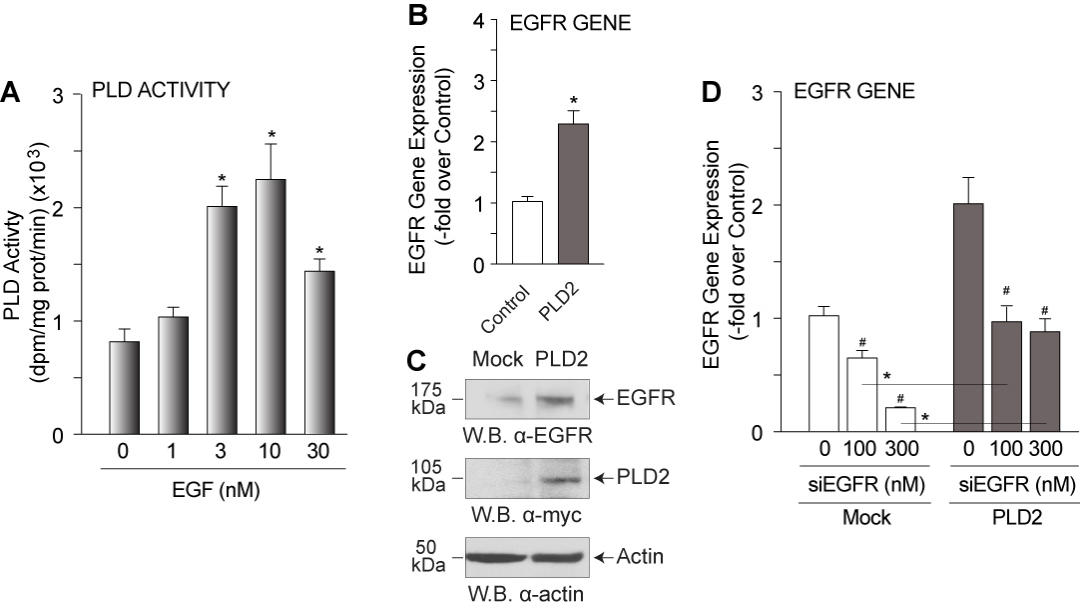
PLD2-EGFR feedback regulation COS-7 cells were mock-treated or transfected with 2 μg of PLD2-WT plasmid DNA and then 48 h post-transfection, cells were incubated with EGF or PA and then were used for PLD lipase activity assay, SDS-PAGE and subsequent western blot analysis or for RNA extraction and QPCR analyses. **A.** Increasing EGF dose (0-30 nM) effect on endogenous PLD lipase transphosphatidylation activity. **B.** QPCR analysis indicating that overexpression of myc-tagged PLD2 positively affected EGFR gene expression compared to mock-transfected control. **C.** Western blot analysis showing an increase in EGFR protein expression upon PLD2 overexpression. **D.** QPCR analysis indicating that PLD2 overexpression reverses the negative effect of silencing EGFR with double stranded siRNA and returns EGFR gene expression to basal levels. The (*) symbols above bars denote statistically significant (*P* < 0.05) ANOVA increases between samples and controls. The (#) symbols above bars denote statistically significant (*P* < 0.05) ANOVA decreases between samples and controls.

There are a number of currently known signaling pathways that exist in the cell that could account for the transport of PA in the cell. We have hypothesized that one mechanism that could shuttle PA around and throughout the cell is that of receptor trafficking that occurs in cells *in viv*o as a result of growth factor-dependent stimulation. As EGF is a tried and true way of stimulating cells *via* PLD, we wanted to investigate if there is a feedback mechanism. Stimulation of mammalian cells with increasing EGF concentrations yielded a significant increase in endogenous PLD transphosphatidylation activity (Figure 1A), suggesting that increased PLD activity could occur a result of an increase in PLD in the cells or as a result of a positive effect on EGF signaling. To address this, we determined the effect of PLD2 overexpression in mammalian cells on EGFR gene expression. Changes in EGFR due to PLD2 overexpression were manifested as a significant increase in EGFR gene expression (Figure 1B) and EGFR protein expression (Figure 1C). When EGFR was silenced using siRNA specific to EGFR, PLD2 overexpression rescued EGFR gene expression to basal expression levels compared to mock-treated controls (Figure 1D). These data suggest that increased PLD2 as a result of EGF stimulation in the cell in turn has a positive effect on EGFR expression.

### PLD aids in and increases cellular EGFR internalization

**Figure 2:**
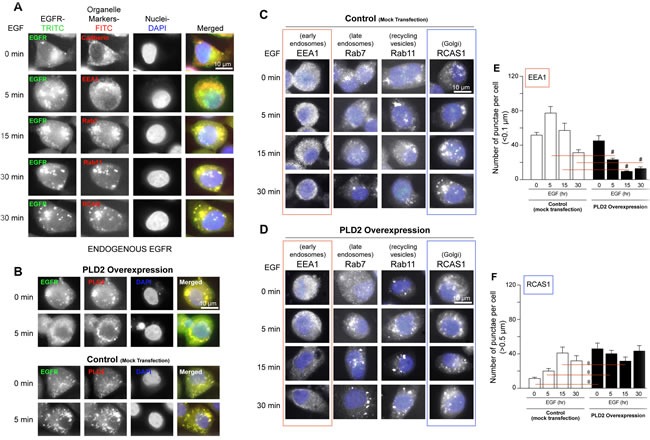
Enhanced EGFR endocytosis due to PLD2 overexpression **A.** Endogenous EGFR intracellular localization following EGF stimulation. MTLn3 cells were serum-starved and then treated with 10 nM EGF for the times indicated. EGFR was stained with α-EGFR-FITC IgG antibodies (left column of panels), while proteins specific to the different organelles (cell membrane, Cadherin; early endosomes, EEA1; late endosomes, Rab7; recycling vesicles, Rab11 or Golgi, RCAS1) were stained with anti-TRITC IgG antibodies (2^nd^ column from the left). Nuclei were stained with DAPI (blue) (2^nd^ column from the right). **B.** After PLD2 overexpression, EGFR was stained with α-EGFR-FITC IgG antibodies (green staining), while antibodies specific to PLD2 were stained with anti-TRITC IgG antibodies (red staining). Nuclei were stained with DAPI (blue). Merged images of overlaid FITC, TRITC and DAPI images are shown in right panels. Shown are images representative of *n* = 3 independent experiments, with visualization of 5 fields in each condition, with similar results. **C.**-**D.** Cells were serum-starved and then treated with 10 nM EGF for the indicated times. Proteins specific to different cellular organelles were as in the previous panel. TRITC images were inverted from color to black/white and then blue DAPI images were merged together using Adobe PhotoShop (all panels as shown). For (A-D), images are representative of *n* = 3 of separate experiments after visualizing 5 separate fileds. Scale bar = 10 μm. **E.**-**F.** Quantification of the total number of punctae per cell (>0.1 μm) for either early endosomal EEA1 (E) or Golgi RCAS1 (F) staining following EGF treatment in the absence or presence of PLD2 overexpression, respectively. The (*) symbols above bars denote statistically significant (*P* < 0.05) ANOVA increases between samples and controls. The (#) symbols above bars denote statistically significant (*P* < 0.05) ANOVA decreases between samples and controls.

Since there is a positive effect of PLD on EGFR expression and vice-versa, as shown in the previous figure, we hypothesize that there is a putative transport regulation of EGFR in the cell by PLD. In order to study this, we determined where EGFR localizes in the cell using known intracellular organelle markers and determined the level of EGFR trafficking and endocytosis, as a result of EGF stimulation. For immunofluorescence microscopy, we used MTLn3 breast cancer cells as an *in vivo* model of EGFR-mediated trafficking, as these breast cancer cells contain upwards of ~5 × 10^4^ EGF receptors per cell [9]. As shown in Figure 2A, the internalization of endogenous EGFR progressed from the cell membrane at time 0 based on Cadherin staining (top panel), to early endosomes and nuclei at 5 min based on EEA1 and DAPI staining (second panels from top), to late endosomes based on co-localization with Rab7 staining at 15 min (middle panels) and finally to recycling endosomes and Golgi based on co-localization with Rab11 and RCAS staining at 30 min (bottom two set of panels). EGFR was localized more to the nucleus at early times of EGF stimulation (~5 min) when compared to later times of EGF stimulation (15-30 min). EGFR co-localization with Rab7 (late endosomes), Rab11 (recycling vesicles/endosomes) and RCAS (Golgi) was stronger at later times of EGF stimulation when compared to earlier times of EGF stimulation. These data indicate that EGFR was quickly internalized to the cytoplasm and then towards the nucleus in a short period of time (~ 5 min).

### PLD2 increases vesiculation

To further investigate a possible link between EGFR and the PLD2 pathway, we transfected PLD2-WT plasmid into the MTLn3 cells and used these PLD2 overexpressing cells for similar microscopic studies as in Figure 2A. Based on the co-immunofluorescence signals (yellow signal in merged images) shown in Figure 2B, both endogenous PLD2 and overexpressed PLD2 co-localized with endogenous EGFR *in vivo* following 5 min EGF stimulation. The accumulation of EGFR was more pronounced at 5 minutes in PLD2-overexpressing cells (Figure 2B, bottom panels) compared to control, mock-transfected cells (Figure 2B, top panels). Additionally, control cells showed localization of EGFR in smaller vesicles and in or on the nucleus, while PLD2-expressing cells showed diffuse and larger punctuated formations (>1 μm) almost exclusively outside the nucleus (Figure 2B). This data suggests PLD2 protected EGFR from degradation in vesicles and increased diffuse cytoplasmic EGFR receptor mass. As a result of EGF stimulation, EGFR progressed at time 0 from the cell membrane (Cadherin) to early endosomes at 5 min (EEA1), then to late endosomes (Rab7) at 15 min and finally to recycling endosomes (Rab11) and Golgi (RCAS) at 30 min. There is also a near nuclear localization of EGFR after 5 min of EGF stimulation. Collectively, these immunofluorescence data support an alteration of EGFR cellular distribution occurred following PLD2 overexpression.

Additionally, global EGFR-mediated endocytosis seemed to be enhanced as a result of PLD2 overexpression with prolonged localization/internalization with late endosomes, recycling vesicles and Golgi after EGF stimulation compared to mock-transfected cells (Figure 2C-2D). A comparison between Figure 2E-2F indicates that a larger number of punctae in the Golgi (RCAS1) are formed as a function of both time in EGF stimulation and PLD2 overexpression. These results show that PLD2 shifts vesiculation from early endosomes to prolonged internalization in late endosomes through Golgi.

### Exogenous PA enters the cell and forms vesicles in a time dependent manner

**Figure 3:**
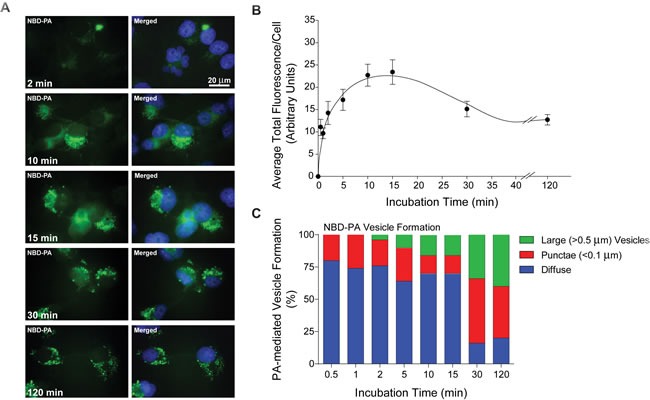
Fluorescent NBD-PA enters cells and forms vesicles **A.** COS-7 cells were mounted onto glass coverslips and then incubated with 30 nM NBD-PA at 2-120 min incubation times. NBD-PA appears green similar to FITC and nuclei were stained blue with DAPI. Scale = 20 μm. **B.** Graph of average total fluorescence per cell over the NBD-PA time course of 0.5 to 120 min. **C.** Stacked vertical bar graph representing NBD-PA-mediated vesicle formation over the time course of 0.5 to 120 min. Green, larger vesicles (>0.5 μm). Red, punctae ( < 0.1 μm). Blue, diffuse localization throughout. Each time point is at least *n* = 4 and is represented as mean + SEM.

Next, we investigated whether or not EGFR endocytosis in response to EGF stimulation occurred as a result of increased vesiculation induced by PLD2's catalytic product, PA. To simulate what would happen inside a cell with a high PA (mimicking overexpression/activation of PLD2) intracellular environment, we chose to incubate the cells with a fluorescently-labeled PA and then visualize PA-mediated vesicle trafficking. First, to provide evidence that exogenous incubation of mammalian cells with PA actually resulted in entry of the PA into the cell, we used PA that contained a [(7-nitro-2-1,3-benzoxadiazol-4-yl)amino]hexanoyl fluorescent green tag (see Material and Methods) [34–36]. To determine if extracellularly added NBD-PA entered into mammalian cells through the plasma membrane, 30 nM NBD-PA was added to COS-7 cells that were mounted on glass coverslips and then incubated for increasing amounts of time (2-120 min). Cells were then fixed and the green fluorescence of the NBD-PA visualized using immunofluorescence microscopy with DAPI-stained nuclei as an internal reference point. At shorter incubation times (Figure 3A-3B), the fluorescence was seen as a diffuse green throughout the cell (2 min). As time increased (10-15 min), NBD-PA small ( < 0.1 μm) punctae vesicles were observed that started to accumulate near the nucleus and possibly in the Golgi. At later times (30-120 min), more punctae and even larger vesicles (>0.5 μm) formed in the cells, which appeared to migrate away from the initial perinuclear localization points.

To determine if this increase in vesiculation as a result of increased NBD-PA incubation time was valid, 50 cells at each time point were observed and the average total fluorescence signal per cells (Figure 3B) and the percentage of PA-mediated vesicle formation (Figure 3C) were determined. As shown in Figure 3B, there was a biphasic effect of NBD-tagged PA incubation in mammalian cells with peaks at 2 min and at 15 min. The fluorescence localization of NBD-PA shown in Figure 3A was observed in three distinct intracellular locations: diffuse throughout the cytoplasm, punctae (small vesicles) and large vesicles.

This data was then also plotted in the stacked bar graph shown in Figure 3C, which shows that there was a concomitant increase in vesiculation both of the smaller punctate vesicles and larger forms, as NBD-PA incubation time increased. The largest increase in vesicle formation occurred at the two longest times of incubation, 30 min and 120 min with ~20% of the cells still showing only diffuse cytoplasmic localization of the NBD-PA. This suggests that exogenously added PA can indeed cross the plasma membrane into the cell, but also that this PA forms vesicles once inside the cell similar to that observed with PLD2 overexpression (Figure 2). Our data complements previously observed PA dynamics using a biosensor [37].

### Exogenous PA containing vesicles are directed towards the nucleus

Next, we investigated what type of vesicles PA formed and also where these vesicles localized to inside the cell to see if this was the mechanism by which EGFR could be internalized into the cell after EGF stimulation. As shown in Figure 3A, the migration of NBD-PA into the cell and its initial perinuclear concentration, subsequent formation of vesicles and then apparent migration away from this cellular location suggests that NBD-PA migrated to the Golgi for packaging in recycling vesicles for transport around or out of the cell.

**Figure 4:**
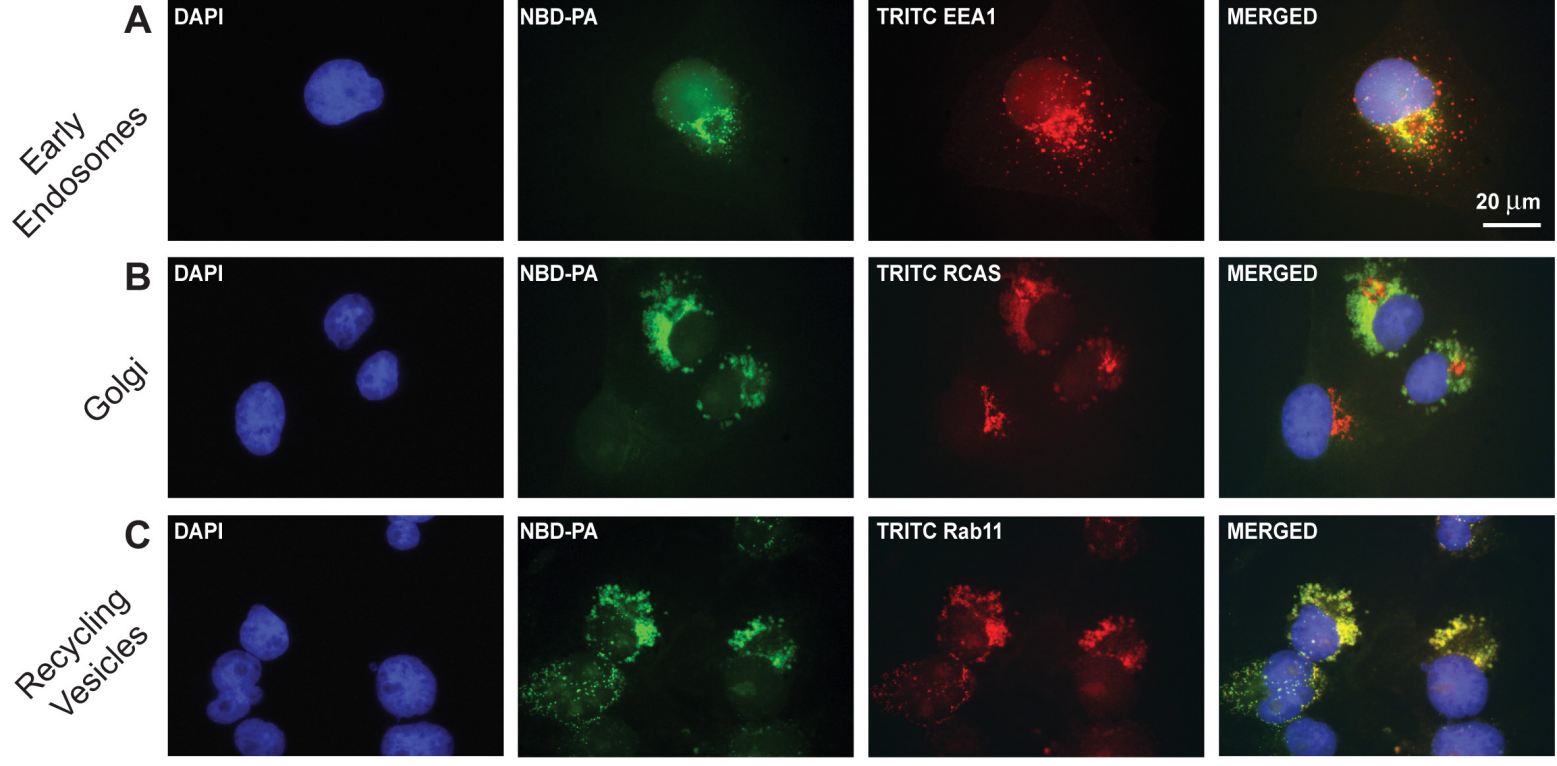
Fluorescent NBD-PA co-localizes with EGFR, the Golgi, early endosomes and recycling vesicles c for 30 min and then cells were fixed using paraformaldehyde. Coverslips were incubated with antibodies for following intracellular marker to measure localization of NBD-PA with the relevant intracellular marker. **A.** early endosomes by staining for TRITC-EEA1, **B.** Golgi by staining for TRITC-RCAS, **C.** and recycling vesicles by staining for TRITC-Rab11. Nuclei were stained blue with DAPI. Nuclei stained blue with DAPI. Scale of A-F = 20 μm.

How NBD-PA ultimately traveled into and throughout cells was investigated. To investigate this process, cells were again incubated in 30 nM NBD-PA but for a set time point (30 min) and co-localization of NBD-PA with TRITC-labelled antibodies specific to several intracellular markers (EEA1 for early endosomes, RCAS for Golgi or RAB7 for recycling vesicles) were used for immunofluorescence microscopy (Figure 4). First, we probed NBD-PA-treated cells mounted on and fixed onto glass coverslips with antibodies for the early endosome marker 1 (EEA1). Co-localization of NBD-PA was seen with early endosomes indicating transport of extracellular PA into the cell (Figure 4A). Secondly, we determined that NBD-PA co-localized with the Golgi using an antibody for the Golgi marker RCAS. As seen in Figure 4B, NBD-PA migrated to and concentrated in the Golgi, which then packaged the NBD-PA into recycling vesicles, as detected using a Rab11 antibody (Figure 4C). These data are evidence of a directed pathway of PA entering the cell and migrating to the Golgi where it induced increased vesiculation, much like endogenous PA has been previously found to be packaged into recycling vesicles for trafficking within the cell [38].

**Figure 5:**
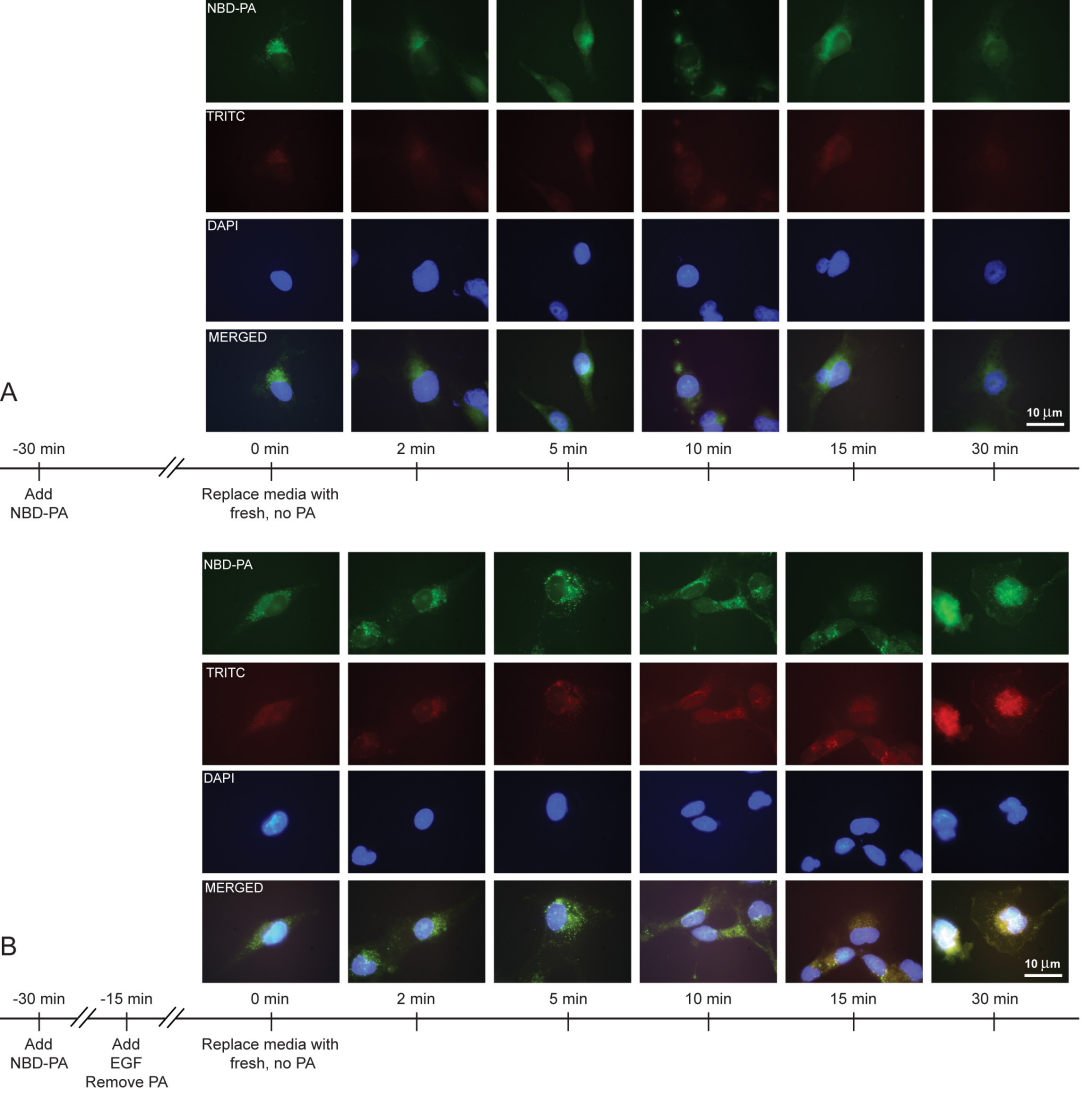
PA vesicles co-localize with endogenous EGFR **A.** COS-7 cells were mounted onto glass coverslips and then incubated with 30 nM NBD-PA for 30 min. Media containing the PA was removed, cells rinsed with 1X PBS, and then fresh media added. Cells were then fixed using paraformaldehyde at the indicated time points of 0 to 30 min. Nuclei were then stained with DAPI. **B.** COS-7 cells were mounted onto glass coverslips and then incubated with 30 nM NBD-PA for 30 min and then 15 min in 30 nM EGF. Media containing the PA was removed, cells rinsed with 1X PBS, and then fresh media added. Cells were then fixed using paraformaldehyde at the indicated time points of 0 to 30 min. Coverslips were incubated with antibodies for EGFR localization by staining for TRITC-EGFR and nuclei stained with DAPI.

To further visualize PA after packaging into recycling vesicles, we again incubated cells for 30 min in 30 nM NBD-PA, but this time the PA-containing media was removed. Figure 5A demonstrates that PA staining was diffuse throughout the cytoplasm at earlier time points (0 to 5 min) but quickly localized to the perinucleus. Greater than 10 min, PA diffused back into the cytoplasm away from the nucleus.

Next, we wanted to determine if EGFR was contained in these PA vesicles, specifically at the later time points when PA was shuttled out of the cell.

In order to demonstrate that EGFR co-localized with these PA vesicles particularly in the stimulated condition we are interested in, we incubated cells first with PA followed by EGF stimulation. At later time points >10 min, PA vesicles co-localized with endogenous EGFR and by 30 min both the PA and EGFR were concentrated in the plasma membrane (Figure 5B). These findings suggest PA-mediated vesiculation as a possible mechanism regulating EGFR's internalization and subsequent return to the membrane, which is similar to results shown in Figure 2 that represent PLD2 co-localization with EGFR.

### PLD in combination with PA affects EGFR and nuclear receptor expression

As stated initially, PLD2 increases EGFR expression, and the product of the PLD reaction, PA, increased internalization of vesicles. We have also seen in Figures 3, 4 and 5 a greater accumulation of PA in vesicles around the nucleus and that this surge in PA led to an increase in EGFR expression, leading us to investigate the nuclear mechanism by which PA increases EGFR expression. PA localizes to the nucleus and interacts with nuclear receptors [39–41]. We have shown previously that PA alters the secondary structure of PPARα [41]. Conserved regions on the EGFR promoter are bound by putative response elements on nuclear receptors (NR), members of the Peroxisome Proliferation Activated Receptor (PPAR) family PPARα, liver X-receptor α (LXRα) and retinoid X receptor α (RXRα).

**Figure 6:**
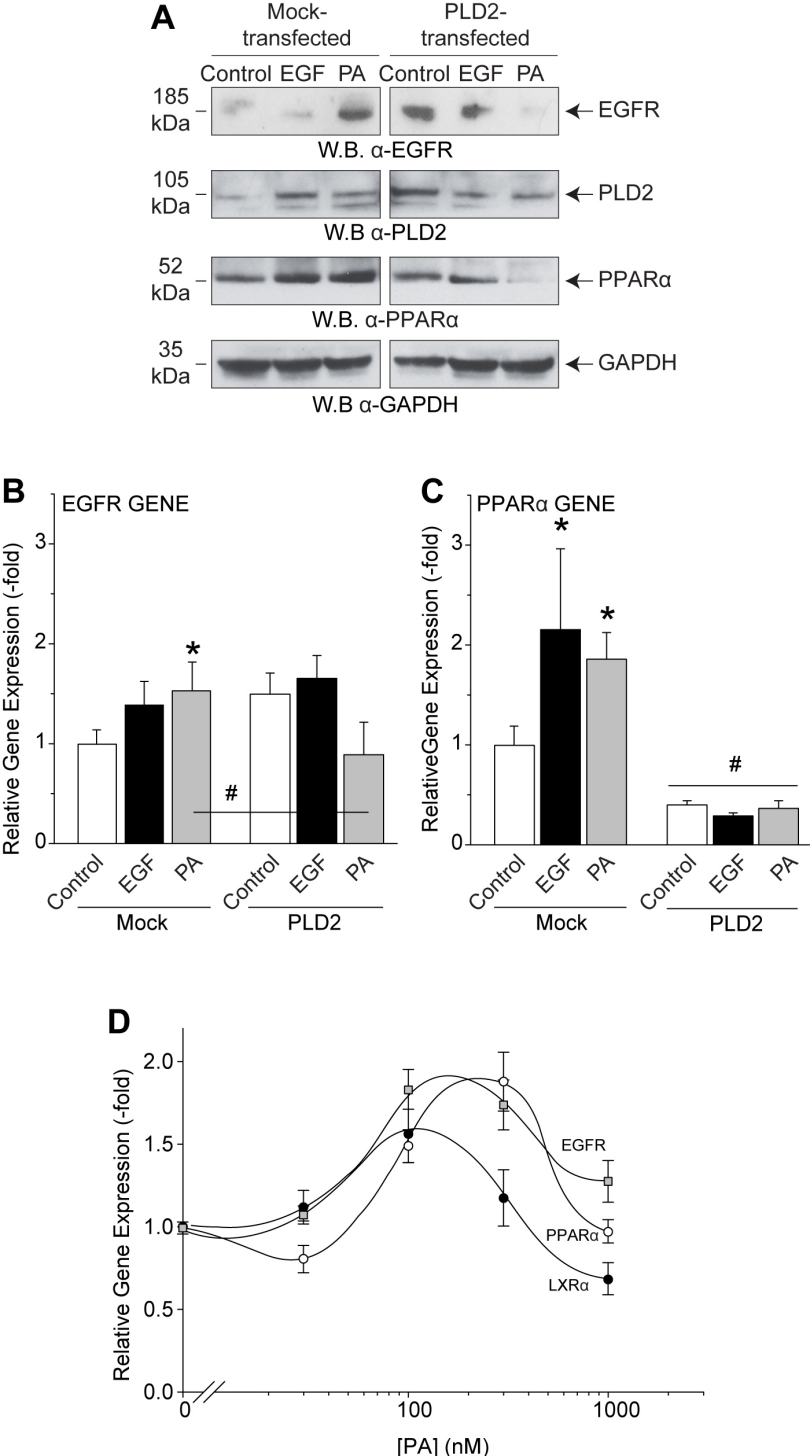
PA targets EGFR and also the nuclear receptors of the PPARα family **A.** Western blot analysis showing the effects of 10 nM EGF or 300 nM PA or increasing concentrations of PA treatment on protein expression of EGFR and upstream nuclear receptor and transcription factor PPARα in the absence or presence of PLD2 overexpression. **B.**-**C.** QPCR analyses of samples detecting either EGFR (B) or PPARα (C) gene expression. **D.** QPCR analyses of samples detecting EGFR, PPAR or LXR gene expression with increasing amounts of PA treatment (0-1000 nM). The (*) symbols above bars denote statistically significant (*P* < 0.05) ANOVA increases between samples and controls. The (#) symbols above bars denote statistically significant (*P* < 0.05) ANOVA decreases between samples and controls.

As seen by Western blot to detect relative protein levels, PA increased EGFR in mock-transfected mammalian cells (Figure 6A); likewise, PLD2 overexpression increased EGFR expression, similar to Figure 1. Interestingly, we discovered that the combination of both PA treatment and PLD2 overexpression had an inhibitory effect on EGFR protein expression. We hypothesized that if PA is at a very high-level in the cell (i.e. transfection for two days of the enzyme that synthesizes PA plus further addition of exogenous PA), then expression of genes/proteins is turned off as a result of a negative feedback. These conditions shut down the trafficking of EGFR.

As this reversal at high PA concentrations is not specific to EGFR alone, we also looked at other genes, specifically both PLD2 and the PPARα nuclear receptor (Figure 6A), which are is known to be activated by EGF signaling [41]. Data shown in Figure 6A-6C representing protein expression and gene expression indicates that even though 300 nM PA alone increased the expression of genes in general (Figure 6B-6C), the combination of both PLD and exogenous PA yielded the opposite effect, that of decreased expression of genes. This suggests that overexpression of PLD2 decreased the threshold of PA's action on gene and protein expression.

This dichotomous effect is also shown in Figure 6D whereby PA treatment biphasically affected the expression of another PPAR family nuclear receptor (LXRα). There was more gene expression of EGFR, PPARα and LXRα at lower PA concentrations, while higher PA concentrations reversed and lowered expression of the respective genes. This suggests that PLD2 in combination with its product PA regulates EGFR and PPARα/LXRα gene expression differently than PLD2 or PA alone presumably due to the overabundance of PA accumulating in the nucleus. We also report for the first time that PPARα/LXRα form heterodimers that bind to Responsive Elements (RE) in the EGFR promoter.

### PA has the capability to biphasically alter PPARα binding to DNA

**Figure 7:**
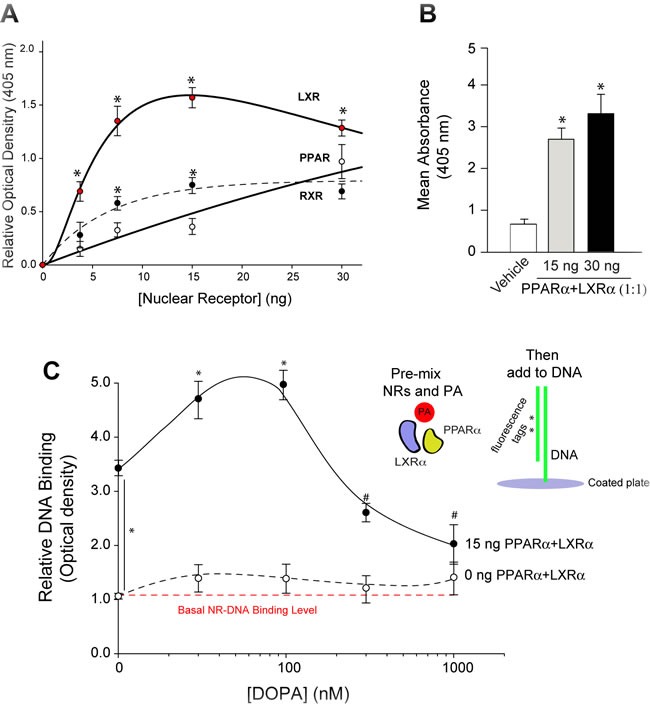
PPAR family Nuclear Receptors (NR) bind to the EGFR promoter **A.** DNA-binding capacities of increasing amounts of PPAR family nuclear receptors (PPARα, LXRα or RXRα) to a synthetic EGFR promoter DNA substrate (*see Supplementary Figure S1*). **B.** Negative control samples that measured the effect of NR (1:1 PPARα+LXRα) binding to the dsDNA substrate in the absence of dioleoyl-PA. Results expressed in terms of mean absorbance at 405 nm + SEM. Results are presented in terms of mean relative optical density at 405 nm + SEM. Experiments were performed in triplicate for *n* = 3. The (*) symbols above bars denote statistically significant (*P* < 0.05) ANOVA increases between samples and controls. **C.** Increasing dioleoyl-PA concentrations were pre-incubated with the PPARα+LXRα heterodimers and then subsequently were added to the EGFR promoter-containing DNA-BIND ELISA plate for completion of the assay. Results are expressed in terms of normalized relative DNA binding + SEM. Shown are images representative of *n* = 5 for each condition with similar results.

The effect of PA on nuclear receptor function on regulating EGFR expression was determined next. To do this, we measured the binding affinities of NRs to the EGFR promoter in the presence of PA. Using the EME assay (EMEA) as described in the Materials and Methods and according to the methodology presented in Supplementary Figure 1S, we measured binding of the NRs to a small consensus sequence on the EGFR promoter. In Figure 7A, the three nuclear receptors (LXRα, RXRα and PPARα) bind to the EGFR promoter in a concentration-dependent manner. It also shows that both LXRα and PPARα have greater binding affinities to the DNA than that of RXRα. Knowing that these NRs have physiological functions as heterodimers, we measured the binding of heterodimer LXRα-PPARα and found a significantly robust binding of the NR heterodimer to the EGFR promoter when compared to the negative control sample (Figure 7B).

Next, the experimental setup for EMEA was conducted using nuclear receptors that were pre-incubated with increasing concentrations of PA before addition of the lipid-protein mixture to the DNA-coated plate (Figure 7C). We observed a robust fluorescence signal indicating NR-EGFR promoter DNA binding in the presence of the heterodimeric PPARα-LXRα. This signal was further augmented with PA, as long as PA was < ~100 nM. Higher PA concentrations had a detrimental effect on binding and reduced binding of the NRs to the EGFR promoter to that of the controls, effectively indicating that no binding of NR to the EGFR promoter occurred at those concentrations.

Extrapolating these findings to the physiological observation of our study, Figure 6 serves to indicate that PA had a dual effect on EGFR expression *via* PPARα where low PA concentrations enabled NR binding to the EGFR promoter and high PA concentrations impeded NR binding to the EGFR promoter. The latter could explain the observed effects in previous figures whereby EGFR gene and protein expression was decreased in the presence of both PLD2 overexpression and exogenous PA. We posit that this effect seen with PA concentrations >100 nM inside the cell is a strong signal for PA to reverse its mitogenic effect, turn off its positive effect on EGFR expression and, ultimately, shut down the conveyor system to stop providing new EGFR to the cell membrane.

## DISCUSSION

**Figure 8:**
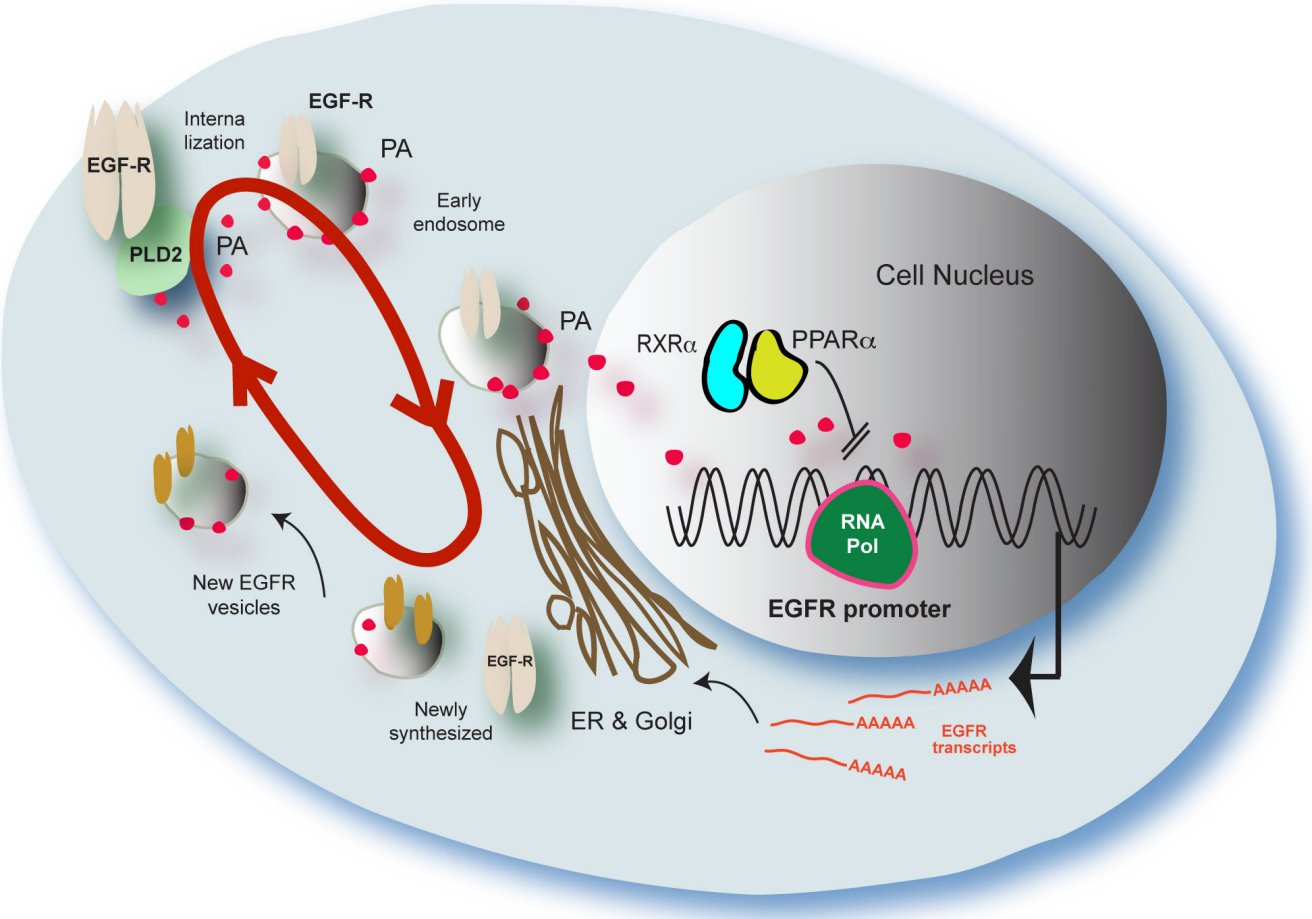
Proposed model to explain results presented in this study The red circular arrow represents the PA “conveyor” transport system described in this study for the firt time that helps with the trafficking of the EGFR form the cell membrane to the nucleus and back (with newly-synthesized receptor). PA also targets PPARα that directly affects the EGFR promoter. The model attempts to explain the complex regulation of trafficking of the EGF receptor upon generation of PA and displacement of nuclear receptors from binding to the EGFR promoter. An increased loop of PA/EGFR trafficking is initiated by binding of EGF to its receptor. Both EGFR and PA are transferred to the nucleus. PA in the nucleus activates gene expression of several genes, as PA is a mitogen. However, it comes to a point in which there is an overabundance of PA in the nucleus, when this occurs, PA negatively shuts down the process. This is done through nuclear receptors (PPAR and LXR) as we show that PA is able to displace NR form DNA promoter of EGR. The trafficking and expression of new protein are shut down.

The model we present (Figure 8) attempts to explain the complex regulation of trafficking of the EGF receptor upon EGF stimulation and also the generation of PA and subsequent displacement of nuclear receptors from binding to the EGFR promoter to thus promote synthesis of new EGFR. The red circular arrow represents the PA “conveyor” that helps with the trafficking of the EGFR from the cell membrane to the nucleus, the Golgi, and back to the membrane (with newly-synthesized receptor). Once activated by EGF, EGFR phosphorylates PLD2 causing an upregulation in PA production. Both EGFR and PA are transferred to the nucleus to influence gene expression, and some to the Golgi for recycling/export back out of the cell. PA in the nucleus activates gene expression of several genes including EGFR, as PA is a mitogen [39]. Newly synthesized EGFR is then shuttled through the Golgi to the plasma membrane through vesicles. As known, lipids play a fundamental role in intracellular trafficking and active transport, particularly in the Golgi and exocytosis [42]. However, it comes to a point in which there is abundant accumulation of PA in the nucleus and/or a loss in EGF signal. When this occurs, PA shuts down this process. This is done through nuclear receptors PPAR and LXR heterodimers, as we show that PA is able to displace NR from the EGFR promoter leading to inhibition of EGFR transcription. The trafficking and expression of new protein is thus effectively decreased or shut down.

This study examined the endogenous mechanisms of regulation of EGFR expression and vesicular trafficking with an aim to determine PLD2 and PA's roles in mediating this process in EGFR signaling. We found that PA was crucial to the transport of the EGFR from the membrane towards the nucleus. The co-localization data for EGFR staining in early endosomes coincides with intracellular elevated levels of PA. The transient nature of PA is reflected by changes in PLD2 expression, localization and activity. This novel work identifies the unexpected result that fluctuations in phosphatidic acid levels in the cell constitute a driving force for an intracellular conveyor that shuttles EGFR from the cell membrane towards the cell nucleus. Furthermore, this PA conveyor initially activates the EGFR promoter through the nuclear receptors PPARα and LXRα. Lastly, newly synthesized EGFR is shuttled back to the cell membrane, which was aided by PA.

Examples of phospholipids cycling intracellularly have been recently described. Platelet derived factor receptor (PDFR), like EGFR is subjected to clathrin-mediated internalization to endosomes, upon activation by its ligand, a pathway that is sensitive to Rab35 activity, and both proteins were constitutively expressed in lysosomes. There is a pool of PI(4,5)P_2_ on endosomal membranes that regulates growth factor sorting and degradations [43], which raises questions about how the membrane architecture is remodeled continuously and has a profound impact on signaling in health and disease. Endomembrane based signaling may not be as prominent as plasma membrane signaling in normal sites. However, GTPase bound RAB35 is internalized in cells to EEAAI and LAMP2 positive compartments, where it drives constitute activation of PI3K/AKT signaling [44]. These observations bring to mind the critical role for Ras in supporting PI3K signaling and trafficking of intracellular membranes.

Lipid-exchange cycles mediate the enrichment of phosphatidylserine (PS) in the cell membrane over the endoplasmic reticulum (ER) where the lipid is produced. Cyclic transport occurs from the origin in the ER thanks to oxysterol-binding proteins (OSBP) (ORP5 or ORP8 in mammals) that bind PS and phosphatidylinositol-4 phosphatase (PI4P) in a mutually exclusive fashion. PS is carried to the plasma membrane in exchange for PI4P that is shuttled to the ER and thus a lipid-protein cycle is maintained [45, 46]. A recycling mechanism is also described in the postsynaptic termini of neurons after clathrin-medicated endocytosis, ultrafast endocytosis and endosomal budding close a cycle [47]. In a model of synaptic vesicles after a rapid internalization to the membrane via endocytosis, the vesicle membrane is diverted to an endosome.

In the present study, not only did PA help the transport of EGF, but also we found that it was important for the regulation of EGFR gene and protein expression. Nuclear receptor activation of the PPAR family of receptors aided PA in this regulation. The data reported herein indicates how the mitogenic and signaling phospholipid PA affected gene transcription of EGFR. Earlier studies have indicated co-overexpression of PPAR NR heterodimers increased EGFR protein expression and more importantly increased EGFR promoter transactivation [41]. We determined the putative regulatory regions on the EGFR promoter sequence for several known transcription factors (specifically PPAR family members) and then tested those regions. We found that EGFR has 9 potential PPAR Response Elements (RE) (Figure 6 and Supplementary Figure 1S). Since the EGFR promoter had more than one potential RE site, we generated 3 synthetic oligonucleotides containing one of 4 different RE sequences, which were upstream of the transcription start site (TSS).

We then tested the ability of PPARα to actually bind to those sequences using EMEA, similar to EMSA but in an ELISA setting, and synthetic duplex peptides of < 50 bp in length with C-terminal single-stranded thymidine linker regions. We determined *in vitro* the binding affinity of PPARα, LXRα and RXRα alone or in combination for the DNA substrates. This enabled us to detect what sequences the transcription factor favored for binding and which region of the promoter was important for binding. Our data suggests that a binding competition occurred between the NRs+DNA and NRs+PA resulting in a biphasic effect of PA on NR heterodimer binding to the EGFR promoter. This shift in binding affected the functionality of PPARα in a dose-dependent manner as shown *in vitro* using EMEA. The effect of PA on this protein-DNA binding interaction enabled us to understand how this phospholipid affected transcription and expression of EGFR.

We hypothesize that accumulation of increasing PA negatively affected EGFR transcription by preventing binding of NR transcription factors (PPAR family members) to the EGFR promoter and resulted in decreased EGFR expression and intracellular trafficking. PPARα-LXRα heterodimers and even PPAR family homodimers along with PA modulated EGFR expression. Accumulation of PA in the nucleus altered EGFR trafficking *via* displacement of nuclear PPAR heterodimers by PA from the EGFR DNA promoter, which effectively regulates the EGFR conveyor.

Localization of increased EGFR in vesicles at the expense of decreased EGFR in the nucleus suggests that PA transports EGFR from the cell membrane to the nucleus and vice versa. We posit that the temporal/spatial localizations for PA enable an intracellular transporter or conveyor that shuttles EGFR from the cell membrane to the nucleus. We found an interconnection between PLD2/PA, EGFR and PPARα, with PA being the glue that maintains a continuous intracellular traffic. Considering that PA has physical properties to influence membrane curvature and its function as a signaling lipid that recruits cytosolic proteins to appropriate membranes, this new study brings PA to the forefront in the complex transport mechanisms inside the cell.

## MATERIALS AND METHODS

### Reagents

Dulbecco's modified Eagle's medium (DMEM) (cat. # SH30243.01) was from GE Healthcare Hyclone (Logan, UT); Opti-MEM (cat. # 11058021) was from ThermoFisher/Life Technologies (Carlsbad, CA), dioleoyl-PA (1,2-dioleoyl-sn-glycero-3-phosphate) (cat. # 840875) was from Avanti Polar Lipids (Alabaster, AL). Transit2020 (cat. # MIR 5400) transfection reagent was from Mirus (Houston, TX). Monoclonal α-DIG IgG alkaline phosphatase antibody (cat. # A1054), p-nitrophenylphosphate (pNPP) liquid substrate system (N7653) and Exonuclease III (E1131) were from Sigma-Aldrich (St. Louis, MO). Custom, synthetic DNA oligonucleotides based on the EGFR promoter were from IDT (Coralville, IA). Purified, recombinant human PPARα (cat. # ab81927) was from Abcam (Cambridge, MA) and purified, recombinant human LXR (cat. # 31122) was from ActiveMotif (Carlsbad, CA). Green fluorescent 1-oleoyl-2-(6-((7-nitro-2-1,3-benzoxadiazol-4-yl)amino)hexanoyl)-sn-glycero-3-phosphate (NBD-PA) (Cat#: 810175) was purchased from Avanti Polar Lipids (Alabaster, AL). For immunofluorescent microscopy, rabbit Rab7 (H-50) (Cat#: sc-10767), rabbit Rab11 (H-87) (Cat#: sc-9020), rabbit EEA1 (H-300) (Cat#: sc-33585), mouse RCAS1 (D-9) (Cat#:sc-398052) donkey anti-mouse IgG-R (Cat#: sc-2300), and donkey anti-rabbit IgG-R (Cat#: sc-2095) were purchased from Santa Cruz Biotechnology (Dallas, TX).

### Cell culture

Rat MTLn3 breast cancer cells were a generous gift from Dr. Jeffrey E. Segall (Albert Einstein College of Medicine); and maintained in α-MEM media supplemented with 10% (v/v) fetal bovine serum. COS-7 cells were obtained from ATCC and maintained in Dulbecco's modified Eagle's medium (DMEM) supplemented with 10% (v/v) fetal bovine serum (FBS). Cells were maintained at 37°C in an incubator with a humidified atmosphere of 5% CO_2_.

### DNA plasmids and transfection

The plasmids used in this study were as follows: pcDNA3.1-mycPLD2-WT, pSG5PPARα, pSG5-RXRα and pSG5-LXRα. Cells were seeded in 6-well plates with an equal number of cells per well. Cells were then allowed to grow for 12-24 h prior to transfection. Plasmid transfection reactions included 1-2 μg of DNA plasmid and 1 μgDNA:2 μl volume of Transit2020 transfection reagent in 200 μl of Opti-Mem Serum-Free media. This transfection reaction was incubated at room temp for 20 min and was subsequently added to the appropriate well in 2 ml of complete media. Cell transfections were 48 h in duration.

### SDS-PAGE and western blot analyses

To confirm the presence of overexpressed protein, we performed SDS-PAGE and western blot analyses of myc-tagged PLD2 overexpressed in COS-7 cells. Two days post-transfection lysates were prepared and samples were analyzed by SDS-PAGE and Western blot analysis to confirm the presence of PLD2 and EGFR proteins in the cell lysates.

### Fluorescent PA (NBD-PA) experimental set-up

Initially, 1 mM NBD-PA was prepared with 1 mg of NBD-PA in 1.4 mL of “Stock Buffer” consisting of 50 mg of Fatty Acid-free BSA per 10 mL of 1x PBS, pH 7.2. This DOPA was then sonicated on ice 2x 4 s each with a 4 s pause in between sonications. “Intermediate 100 μM liposomes” were then made using 25 μl of the NBD-PA stock solution and 225 μl of Cell Starvation Media (DMEM + 0.1% bovine serum albumin), which were then used to prepare the final concentrations of NBD-PA used to incubate cells for the indicated times in various figures.

### Immunofluorescence microscopy

Cells were transfected and plated onto glass coverslips. Forty-eight h post-transfection (if necessary) cells were fixed using 4% paraformaldehyde, permeabilized using 0.5% Triton X-100 in PBS and blocked using 10% fetal calf serum in PBS and 0.1% Triton X-100 (PBS-T). Cells were incubated with a 1:1000 dilution of α-myc-FITC (green) or α-myc-TRITC (red) antibodies in blocking buffer specific for myc-tagged PLD2, washed three times with PBS and then incubated in a 1:2000 dilution of DAPI in PBS. Cells were washed rinsed and air-dried. Coverslips were mounted onto a glass slide using Vectashield mounting media and were then viewed using a Nikon Upright Eclipse 50i Microscope, a Plan Fluor 100x/1.30 OIL objective and FITC, TRITC or DAPI fluorescence filters. Photomicrographs were obtained using a Lumenera Infinity3 digital camera and Infinity Analyze software. The images shown in results are images representative of *n* = 3 independent experiments, with visualization of 5 fields in each condition, with similar results.

### Gene expression measurement by quantitative real time RT-PCR (qRT-PCR)

Reverse transcription coupled to qPCR was performed following published technical details [48]. Total RNA was isolated from cells with the RNeasy minikit according to the manufacturer's protocol (Qiagen, Valencia, CA). RNA concentrations were determined using a NanoDrop and samples were normalized to 50ng/μl RNA. Reverse transcription was performed with 210 ng RNA, 210 ng random hexamers, 500 μM dNTPs, 84 units RNase OUT and 210 units of SuperscriptII reverse transcriptase and incubated at 42°C for 55 minutes. qPCR reactions were run with 100 ng total input RNA, 1 μl of PLD1 or PLD2 gene expression assay (FAM-labeled) multiplexed with the housekeeping gene (β-Actin) (FAM-labeled) with the final concentrations being 200 pmol and 400 pmol for the primers and probe, respectively. Primers and fluorescent probes were synthesized by Applied Biosystems. qPCR conditions for the Stratagene Cycler were: 50°C for 2 min, 95°C for 10 min and then 40 cycles of the next 3 steps: 30 sec at 95°C, 1 min at 60°C, and then 30 sec at 72°C. The “cycle threshold” Ct values were arbitrarily chosen from the linear part of the PCR amplification curve where an increase in fluorescence can be detected >10 S.E.M. above the background signal. ΔCt was calculated as: ΔCt = Avg. PLD Ct - Avg. Housekeeping Ct, and gene -fold expression was calculated as 2^−(ΔΔCt)^ = 2^−(experimental condition ΔCt - control ΔCt)^.

### Lipid preparation

1,2,-dioleoyl phosphatidic acid (dioleoyl-PA) from Avanti Polar Lipids (Alabaster, AL) was prepared from powder in “stock buffer”: PBS/0.5%BSA (50 mg de-lipidated BSA per 10 ml of 1x PBS) pH = 7.2, with a final concentration of lipids of 1 mM. This solution was sonicated on ice (at medium setting): once for 4 sec; kept on ice for 4 sec, and this cycle was repeated twice more and extruded (Avanti Polar Lipids). Lipids were kept on ice, overlaid with N_2_ in the tubes, tightly caped, and stored at 4°C, protected from light in a desiccator. An intermediate dilution (10 μM) was prepared on the day of the experiment in HBSS+HEPES (0.24 g HEPES/100-ml bottle of HBSS), 0.5% BSA, pH to 7.35. Lipids were added (drop-wise) to the cells (30 μl per 1 ml of cells) for a final concentration of 300 nM unless otherwise indicated.

### *In vitro* PPARα binding to dsDNA promoter and exonuclease-mediated ELISA-like (EMEA) assay

A schematic detailing this entire process can be found in Supplemental Figure 1S. PPARα binding to the EGFR promoter (Supplemental Figure 1S-A) was performed by an exonuclease-mediated enzyme-linked immunosorbent assay (ELISA)-like assay (EMEA) with purified recombinant PPARα, according to the method described in [49]. The oligo sequences are listed in Supplemental Figure 1S-B. For example, the sense oligo for DNA Substrate #3 was 5′-TTCCAAGAGCTTCACTTTTGCGAAGTAATGTGCTTCACACATTGGCT(T)_14_-NH2-3′; and the antisense oligo for DNA Substrate #3 was 3′ - AAGGTTCTCGAAGTG AAAACGCTTCATDACACGAAGDGTGTAACCGA - 5′. In bold-face type is the putative binding site for PPARα; in bold and underlined are the two digoxigenin (D)-labeled nucleotides. Taking advantage of the (T)_14_ linker, the sense oligo was immobilized inititially to a N-oxysuccinimide ester-coated DNA-BIND plate (ThermoFisher, cat. # 07-200-585) at a concentration of 25 pmol in a 100 μl volume per each well in oligonucleotide binding buffer (50 mM Na_3_PO_4_, pH 8.5, 1 mM EDTA) and washed extensively (Supplemental Figure 1S-C). Five pmol of the antisense oligo was added and a dsDNA was formed as in [50]. Plate-bound DNA was incubated with up to 30 ng/well total of homodimers or heterdimers of the nuclear receptors (PPARα, RXRα or LXRα) (if heterodimers like PPARα+LXRα, then in equal concentrations) for 20 min at 37°C. Then the plate was treated with exonuclease-III for 20 min at 30°C to eliminate the fraction of the DNA probe not bound to PPARα. Exonuclease digestion buffer was 60 mM Tris-HCl, 0.6 mM MgCl_2_, pH 8.0. Protected PPARα-DIG-labeled DNA was detected with enzyme-linked immunoassays for anti-digoxigenin alkaline phosphatase (AP) IgG conjugates and visualized by chemiluminescence using p-nitrophenylphosphate (pNPP) liquid substrate system. EMEA plates were read at 405 nm every 15 min for up to 1 hr total. Negative controls had 30 ng/well BSA instead of PPARα. If PA was used in the reaction, then PA was first incubated and pre-bound to the NRs for 10 min, which was then added to the EGFR promoter that was bound to the DNA-BIND plate for 1 h.

### Statistical analyses

Data presented in the Figures as bars are means + Standard Error of the Mean (SEM) (standard deviation/n1/2, where n is the sample size). Experiments were performed in technical triplicates (for qPCR assays) or technical duplicates (for PLD assay) for *n* = 5 independent experiments. The difference between means was assessed by the Single Factor Analysis of Variance (ANOVA) test, calculated using SigmaPlot version 10 (Systat Software Inc, San Jose, CA). Probability of *p* < 0.05 indicates a significant difference. In the figures, the (*) symbols above bars denote statistically significant (*P* < 0.05) ANOVA increases between samples and controls. The (#) symbols above bars denote statistically significant (*P* < 0.05) ANOVA decreases between samples and controls.

## SUPPLEMENTARY MATERIAL


