# Supplementary material for: A Phosphatidic Acid (PA) conveyor system of continuous intracellular transport from cell membrane to nucleus maintains EGF receptor homeostasis
Source: Oncotarget. 2016 May 31;7(30):47002–17. doi: 10.18632/oncotarget.9685 (PMC5216919; doi:10.18632/oncotarget.9685)
Supplement: Supplementary file 1 [file oncotarget-07-47002-s001.pdf]

A Phosphatidic Acid (PA) conveyor system of continuous intracellular transport from cell membrane to nucleus maintains EGF receptor homeostasis

Karen M. Henkels2, Taylor E. Miller2, Ramya Ganesan2, Brandon A. Wilkins2, Kristen Fite2 and Julian Gomez-Cambronero1,2

1 Center for Experimental Therapeutics and Reperfusion Injury, Brigham and Women Hospital and Harvard Medical School, Boston, MA, USA

2 Wright State University Boonshoft School of Medicine, Department of Biochemistry and Molecular Biology, Dayton, Ohio , USA

Supplementary Material

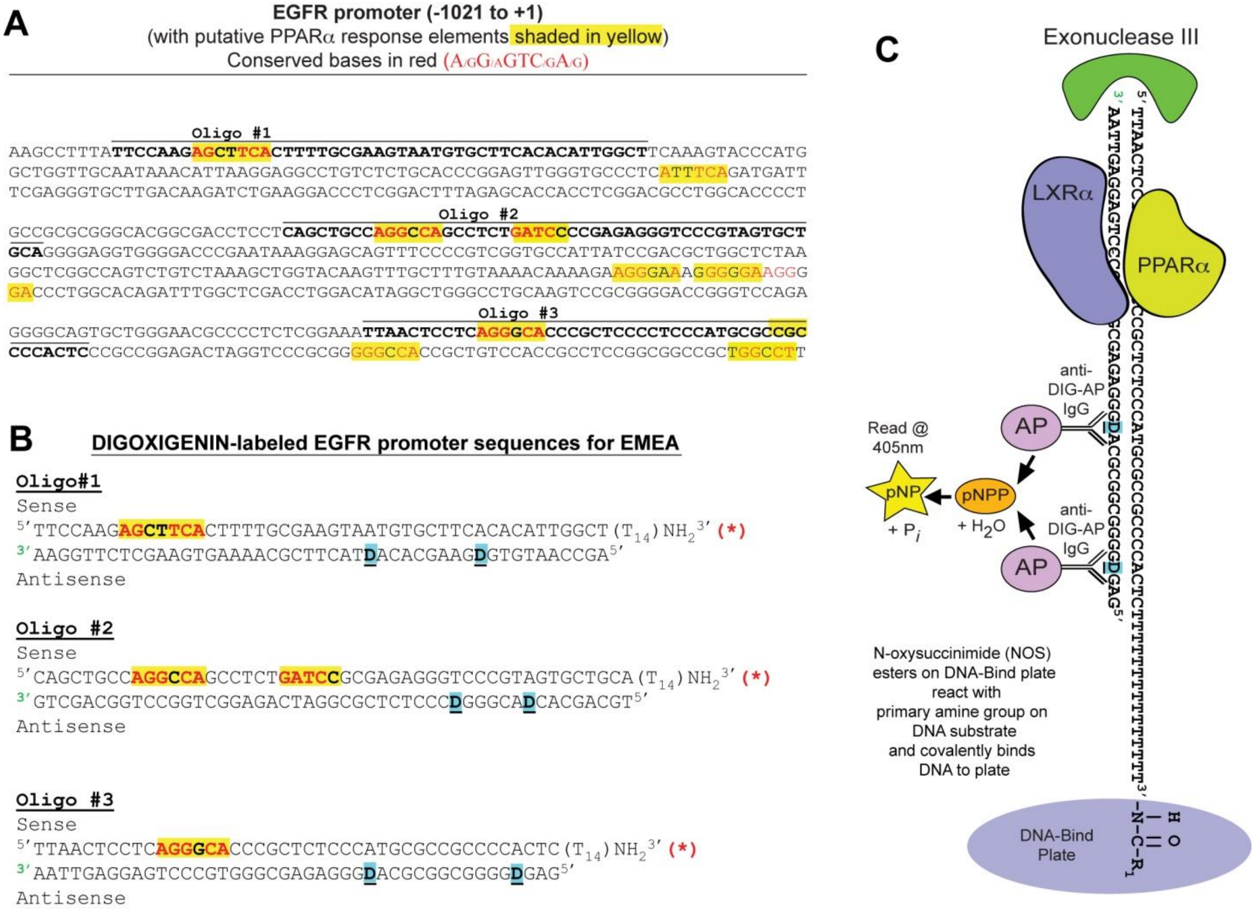

__Supplementary data__. **Figure 1S. PPARα binds to a specific region on the EGFR promoter.** We found 9 putative PPAR response elements on the EGFR promoter with a conserved consensus sequence of A/G-G/A-G-T-C/G-A/G **(A).** We designed and used 3 different duplex oligonucleotides based on the EGFR promoter that contained 1-2 of the putative PPAR response elements (Oligos #1-#3) **(B).** Duplex

DNA substrates based on the EGFR promoter ranging in length from 43-49 bp followed by a single-stranded T_{14} repeat on the 3'-end of the sense strand in combination with a 3'-terminal primary amino group were used for binding to PPAR proteins. These were utilized in an exonuclease-mediated enzyme-linked immunosorbent assay (ELISA)-like assay (EMEA) for detecting the DNA binding activity of PPAR family nuclear receptors to the EGFR promoter. As shown in **(C),** the sense strand of the duplex DNA oligonucleotide substrate contained the putative PPAR response elements based on the EGFR promoter sequence and contained an extensive repeat of thymidines at the 3'-end followed by a primary amino group. This 3'-amino modifier was used to covalently bind the sense oligo strand to a N-oxysuccinimide (NOS)-ester-coated plastic DNA-Bind plate and result in a plate-bound single-strand DNA oligo. This single-strand DNA oligo was then annealed to the complementary antisense strand that contained 2 digoxigenin-modified thymidines for use as a non-isotopic DNA label that was detected with enzyme-linked immunoassays using anti-Digoxigenin IgG antibodies. In our case, we used anti-Digoxigenin-alkaline phosphatase (AP) IgG antibodies because AP could be used to catalyze *p*-nitrophenylphosphate (pNPP) as a substrate to yield phosphate and p-nitrophenyl (pNP) that produced a visible yellow-color change and was quantifiable at 405 nm. If the duplex DNA oligos were bound by any of the PPAR transcription factors, then these substrates would be resistant to Exonuclease III digestion that would degrade the DNA substrates beginning at the 3'-end of the antisense strand. No exonuclease digestion yielded binding of anti-Digoxigenin-AP antibodies to the modified digoxigenins that were used for detection of the protein-DNA binding interaction.
